# Spliced leader RNA silencing (SLS) - a programmed cell death pathway in *Trypanosoma brucei* that is induced upon ER stress

**DOI:** 10.1186/1756-3305-5-107

**Published:** 2012-05-31

**Authors:** Shulamit Michaeli

**Affiliations:** 1 The Mina and Everard Goodman Faculty of Life Sciences, and Advanced Materials and Nanotechnology Institute, Bar-Ilan University, Ramat-Gan, 52900, Israel

**Keywords:** Spliced leader silencing, Unfolded protein response, Programmed cell death, Translocation to the ER, ER quality control

## Abstract

*Trypanosoma brucei* is the causative agent of African sleeping sickness. The parasite cycles between its insect (procyclic form) and mammalian hosts (bloodstream form). Trypanosomes lack conventional transcription regulation, and their genes are transcribed in polycistronic units that are processed by *trans-*splicing and polyadenylation. In *trans-*splicing, which is essential for processing of each mRNA, an exon, the spliced leader (SL) is added to all mRNAs from a small RNA, the SL RNA. Trypanosomes lack the machinery for the unfolded protein response (UPR), which in other eukaryotes is induced under endoplasmic reticulum (ER) stress. Trypanosomes respond to such stress by changing the stability of mRNAs, which are essential for coping with the stress. However, under severe ER stress that is induced by blocking translocation of proteins to the ER, treatment of cells with chemicals that induce misfolding in the ER, or extreme pH, trypanosomes elicit the spliced leader silencing (SLS) pathway. In SLS, the transcription of the SL RNA gene is extinguished, and tSNAP42, a specific SL RNA transcription factor, fails to bind to its cognate promoter. SLS leads to complete shut-off of *trans*-splicing. In this review, I discuss the UPR in mammals and compare it to the ER stress response in *T. brucei* leading to SLS. I summarize the evidence supporting the notion that SLS is a programmed cell death (PCD) pathway that is utilized by the parasites to substitute for the apoptosis observed in higher eukaryotes under prolonged ER stress. I present the hypothesis that SLS evolved to expedite the death process, and rapidly remove from the population unfit parasites that, by elimination via SLS, cause minimal damage to the parasite population.

## Review

### General introduction

In this article, I will discuss a phenomenon discovered in African trypanosomes that was termed spliced leader silencing. Trypanosomes lack conventional transcriptional regulation and thus cannot elicit the unfolded response (UPR), which is based on transcriptional activation [1]. Instead, trypanosomes possess a stress response mechanism that was termed the spliced leader RNA silencing (SLS) pathway [2]. Trypanosome genes are transcribed as polycistronic transcription units, and the generation of mature mRNA requires the processing of the genes by concerted action of *trans-*splicing and polyadenylation [3-5]. In *trans*-splicing, a common exon, the spliced leader (SL), is donated to each mRNA from a small RNA, the SL RNA [3,6]. SL RNA is therefore the most important non-coding RNA in these parasites, and is the only gene in these parasites that harbors a defined polymerase II promoter [7]. In trypanosomes, knockdown of the signal recognition particle (SRP) receptor and factors involved in ER translocation including SEC63 and SEC61, trigger a mechanism that results in silencing of SL RNA transcription, thus freezing the parasite’s ability to produce mRNA [2,8]. SLS is not equivalent to the UPR [9]. However, although the genes that are responsible for UPR in other eukaryotes are absent in trypanosomes, these organisms elicit a clear response as a result of ER stress [9]. Here, I summarize what is known so far regarding the SLS pathway and how it compares with UPR. I present the hypothesis that SLS is used to speed-up the death process elicited by ER stress, thereby providing the parasites with a mechanism to eliminate the unfit organisms from the population. The SLS mechanism supports an altruistic aspect of cell death, in which the death of the unfit individuals enriches the population with the fittest parasites that can sustain a productive infection.

### ER stress in eukaryotes leads to programmed cell death-mechanism and machinery

The endoplasmic reticulum (ER) functions to mediate and control the folding of proteins that traverse its membranes en route to intracellular organelles or the plasma membrane. Eukaryotes have evolved special signaling pathways that are transmitted from the ER to the cytoplasm and the nucleus in response to the misfolding of proteins within the ER [1,10-12].

ER stress results from a number of insults, including exposure to agents that perturb protein folding such as reducing agents, nutrient deprivation, alterations in the oxidative-reduction balance, changes in Ca^+2^ level, and failure to glycosylate proteins [10-12]. The machinery that executes UPR and its regulatory proteins in higher eukaryotes will be described in this review in order to highlight the differences we observed when examining the machinery that reacts to ER stress in trypanosomes.

UPR performs three functions adaptation, alarm and apoptosis. The UPR is first directed to induce folding of the misfolded proteins by the induction of chaperones and at the same time attenuate the damage by reducing the ER load via inhibiting translation, and increasing the degradation of the unfolded proteins. If these steps fail to overcome the catastrophe imposed on the cell, the UPR then induces cellular alarm and apoptosis [13]. The alarm phase is mediated by signaling pathways that leads to the removal of translational block and the down-regulation of the expression and activity of pro-survival factors. After the alarm phase, cells undergo apoptosis (review by [14]).

**Figure 1:**
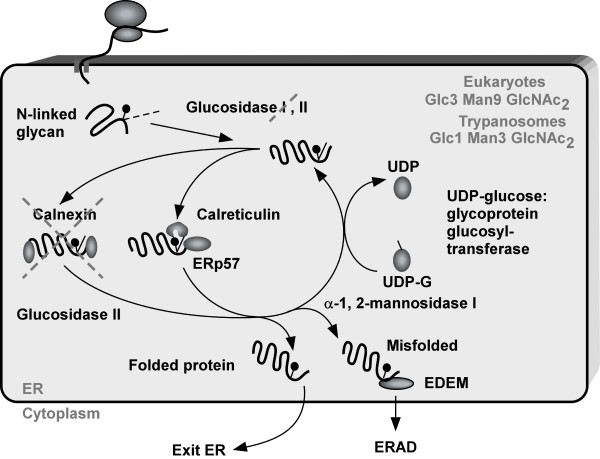
**The ER-quality control.** Upon translocation to the ER the N-glycan is ligated to the nascent chain. Then two glucosidases I and II remove glucose group. The mono-glucosylated glycoprotein then interacts with calnexin/calreticulin. These chaperones recruit the oxireductase ERp57. Cleavage of the last glucose residue by glucosidase II leads to the release of chaperones. At this stage if the protein is properly folded it will exit the ER. The incorrectly folded protein is the substrate of UDP/glucose:glycoprotein glucosyltransferase, which puts glucose back to the misfolded protein. If the protein fails to fold properly even after several cycles, the manose residue is removed by the mannosidase I. This modified glycan is recognized by the (ER degradation enhancing mannosidase-like protein) (EDEM). This targets the misfolded protein for ER-associated degradation (ERAD). The factors missing in trypanosomes but exist in other eukaryotes are crossed.

The ability to sense misfolded proteins relies on a quality control mechanism present in the ER that normally ensures that proteins are properly folded before exiting the ER [15]. Exposed hydrophobic regions, unpaired cysteine residues, or aggregation are markers of unfolded and misfolded proteins. One of the markers for proper folding is also the glycan code [16]. Most of the proteins entering the ER are modified by adding of preassembled oligosaccharides. These are bound by ER lectins calnexin and calreticulin that are associated with the ER oxidoreductase ERp57 [17]. Repeated glycosylation and de-glycosylation cycles ensure misfolded glycoproteins spend sufficient time in the ER to correctly fold (Figure 1). Another important ER chaperone is BiP [18,19], which regulates the activation of the ER transmembrane proteins, the ER stress transducers, described below. BiP is bound to these receptors but in the presence of exposed hydrophobic residues BiP dissociates, allowing their activation [20].

**Figure 2:**
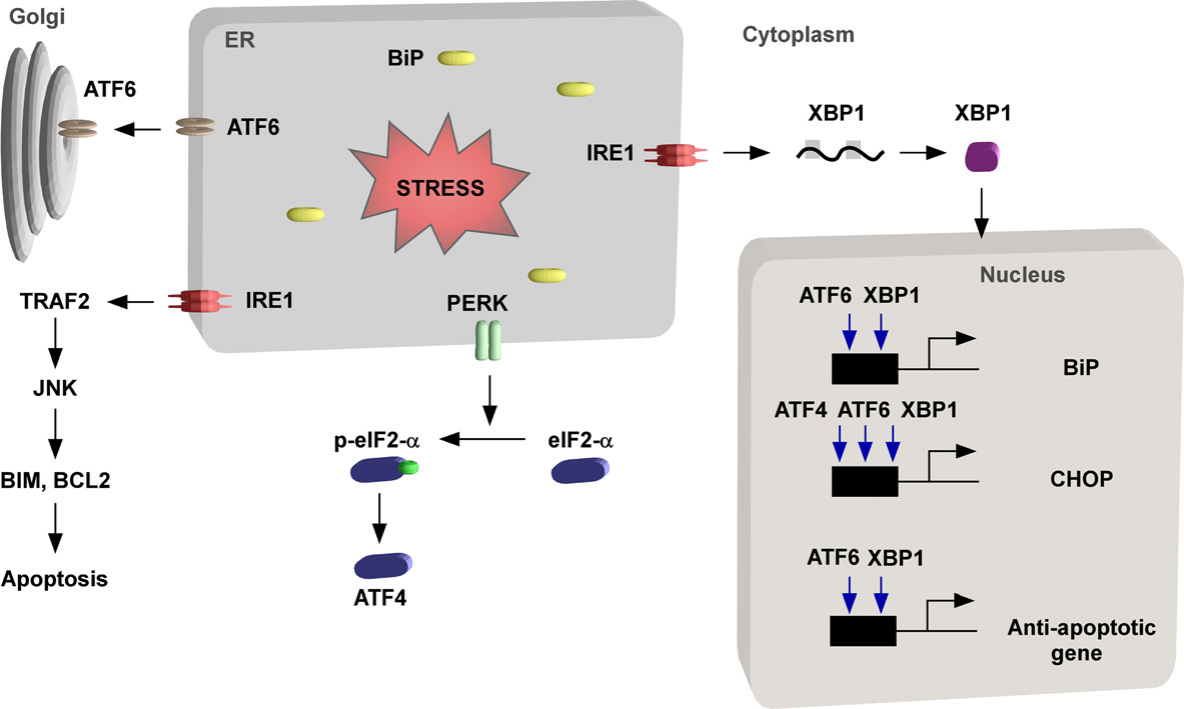
**The two branches of the unfolded protein response.** As a result of accumulation of misfolded proteins in the ER, the unfolded protein response is initiated. Three signal transduction pathways coordinate the pathway and require the dissociation of the ER chaperone BiP. The kinases are: PKR-like kinase (PERK). PERK activation sends both pro-and anti-apoptotic signals but its main function is translation attenuation via phosphorylation of eIF2α which reduces the ER load. ATF6 induces expression of chaperones like BiP, but also the apoptosis factor CHOP. IRE1 is activated and becomes an endonuclease that process the XBP1 mRNA. XBP1 protein is a transcription factor that drives the transcription of both pro- and anti-apoptotic genes. The delicate balance between the protective and destructive branches of the UPR determines if the cell will overcome the stress or will die via the PCD pathway.

In metazoa, the regulators of the UPR include three transmembrane ER-resident proteins, inositol- requirement (IRE1) the (PKR)-like ER kinase (PERK), and the activating transcription factor (ATF6) (Figure 2). The trans-autophosphorylation of IRE1-kinase domain activates its function as an endonuclease that cleaves the transcription factor mRNA XBP1 in metazoans or HAC1 in yeast. After processing of the mRNA and its translation, this transcription factor activates the transcription of UPR target genes including proteins involved in ER-associated degradation (ERAD), the entry of proteins into the ER and protein folding [21] (Figure 2). The gene for XBP1 or HAC1 is also induced under UPR [12]. In *Drosophila,* IRE1 was also shown to be involved in degradation of ER-associated mRNAs [22] and this phenomenon was also observed in mammalian cells and was termed RNA dependent decay or RIDD [23]. Thus, the degradation of ER-associated mRNAs coding for proteins destined to traverse the ER reduces the ER-load. Another essential factor that senses the stress in the ER is ATF6, which is transported from the ER to the Golgi apparatus, where it is cleaved and then translocated to the nucleus to activate genes essential for coping with ER stress, including proteins involved in the anti-oxidant response, chaperones, XBP1, C/EBP-homologous protein (CHOP), a transcription factor that activates target genes including genes involved in growth arrest, oxidases and protein disulfide isomerases (PDI) localized in ER [24]. ATF6 also up-regulates proteins involved in ERAD, which translocate the proteins into the cytoplasm for degradation by the proteasome [25]. ATF6 activation is responsible for transcriptional regulation of pro-survival genes [26] (Figure 2).

The third ER stress transducer is PERK, which is also a ER-localized transmembrane protein whose cytoplasmic portion contains a kinase domain; upon activation, PERK phosphorylates eIF2α thereby globally reducing the load of newly synthesized proteins and decreasing the burden on the ER [27]. However, decreased protein expression is not universal; genes with internal ribosome entry site (IRES) in the 5’ untranslated region bypass the eIF2α translational block [28]. One such protein is ATF4 that drives the expression of pro-survival function such as amino acid transport, redox reaction and protein secretion [29]. However, PERK activation is reversible, due to the action of growth arrest and DNA-damage-inducible protein-43 (GADD34) a phosphatase that dephosphorylate eIF2α. This dephosphorylation coordinates the recovery of eIF2α activity with the transcriptional induction of UPR target genes, enabling their translation [30].

Severely misfolded proteins and protein aggregates might be difficult to bring across the ER membrane via the ERAD system. Cells therefore possess an alternative pathway for protein-degradation, by autophagy. Many of the autophagic factors were shown to be UPR target genes, and important for survival under ER stress [31]. Indeed, under ER stress, ER membranes were shown to become tightly packed into autophagosomes. The main purpose of this process is to sequester the damaged ER.

Together, ATF4, XBP1, and ATF6 govern the expression of a large range of partially overlapping target genes, that their encoded proteins function to alleviate the stress. However, IRE1 signaling also plays an important role in activation of the apoptotic pathway that dominates when all measures to alleviate the stress fail. Phosphorylated, activated mammalian IRE1 interacts with the adaptor protein TRAF2 (tumor necrosis factor receptor) and promotes a cascade of phosphorylation events that activates JUN amino-terminal kinase (JNK) [32]. Once activated, JNK performs a number of functions including the activation of the pro-apoptotic BIM protein [33]. Phosphorylated BIM translocates to the mitochondrial outer membrane, where it promotes cytochrome C release and caspase activation [34]. JNK activation also regulates the activity of anti-apoptotic BCL-2 [35]. Inhibition of BCL-2 and activation of BIM leads to BAX/BAK dependent apoptosis, suggesting that signals initiated from IRE1 participate in the pro-apoptotic branch induced under severe UPR (Figure 2). IRE1 has also been shown to directly interact with the BCL-2 family members BAX and BAK [36]. The activation of BAX and BAK is modulated by one of the IRE1 negative regulator (BI-1). BI-1 is an anti-apoptotic protein that enhances cell survival [37] and BI-1 was shown to interact with IRE1 [38,39]. Another factor that enables cell death is CHOP, whose transcription is induced by eIF2α phosphorylation. CHOP deletion protects against the death of ER stressed cells, and thus its presence may promote cell death [40]. The effect of CHOP might be direct, but it was also noticed that in *chop-/-* cells, the level of GADD43 is reduced, thereby causing a sustained repression of protein synthesis avoiding the synthesis of proteins needed to execute the apoptotic branch of UPR [41,42].

The complex life or death decision for the cell under ER stress becomes evident when inspecting the role and the kinetics of eIF2α phosphorylation. Loss of PERK-mediated eIF2α phosphorylation sensitizes cells to death from ER stress [27]. It was suggested that survival under mild ER stress is maintained because of the instability of the UPR-induced cell death mediators; the level of these proteins become sufficient to induce cell death only under prolonged ER stress [43].

However, in most experiments in which the ER is pharmacologically perturbed, adaptive factors such as chaperones and ERAD components are co-expressed with apoptosis genes with similar induction kinetics. This situation has made it difficult to uncover the mechanisms underlying the distinction between adaptive versus pro-apoptotic ER stress as well as understanding how the transition between these two phases is controlled. Recent reviews present an integrating view on the mechanisms of apoptosis induced by the ER stress in higher eukaryotes [13,14,44].

### ER stress elicited by perturbations of protein translocation induces SLS in *T. brucei*

Trypanosomes are protozoan parasites that diverged very early from the eukaryotic linage. These parasites are known for their non-conventional gene expression mechanism. No polymerase II promoters for protein coding genes were described. The genes are transcribed into polycistronic primary transcripts [3,5,6]. An elegant study showed correlation between the position of histone binding and putative transcription start sites [45], and recently RNA-seq was used to map additional transcription start sites [4]. However, no defined promoters in the strand-switch regions were identified to date [4]. It is therefore believed that gene expression in these parasites is regulated primarily post-transcriptionally at the level of mRNA degradation and translation; the signal that dictates this regulation is confined to the 3’ UTR [46,47]. Alternative *trans-*splicing was also recently suggested as a mechanism underlying differential gene expression of the parasite in its two hosts [5,48,49].

In the absence of transcriptional regulation for individual genes, it could be predicted that trypanosomes may not have a mechanism analogous to UPR. It was also argued that trypanosomes may not need to have a UPR response, because these parasites propagate under homeostasis in the host [50]. Indeed, bioinformatic searches failed to detect IRE1 or XBP1 homologues, which are the key factors in the UPR response, as described above.

As outlined above the mechanism to respond to UPR and eventually dispose of misfolded proteins are well characterized in mammalian cells and yeast. Folding within the ER, mediated by chaperones, protein disulfide isomerase, cycles of glycosylation and de-glycosylation leading to either productive export, or retro-translocation to the cytoplasm for degradation were described above (Figure 1). Retro-translocation is associated with ubiquitylation and proteosomal degradation [51]. In the ER, the cycle of quality control requires BiP, PDI, calencin/calreticulin, glucosidases and a group of mannose-binding proteins (EDEM) that recognize processed N-glycans (Figure 1). Interestingly, trypanosomes lack the Glc_3_Man_9_GlncNAc_2_ that is added to nascent chains of proteins entering the ER, but uses Glc_1_Man_9_GlcNAc_2_ instead. In addition, the parasites encode only for a single glucosidase II but not I [52]. *T. brucei* encodes for a calreticulin orthologue, but lacks calnexin, suggesting a somewhat simpler machinery for ER-quality control than the one found in other eukaryotes [53] (Figure 1). Knocking-down ER-resident proteins involved in this control such as calreticulin, ER glucosidase II, EDEM, the oxireductase ERp72, and ER57p-like protein resulted in defects in proliferation, aberrant morphology, swollen ER, suggesting the presence of ER quality control in these organisms [54]. The presence of such ER quality control in the ER but the absence of a of conventional UPR machinery encouraged me to investigate possible regulation at the first step of entry of proteins to the ER in trypanosomes.

**Figure 3:**
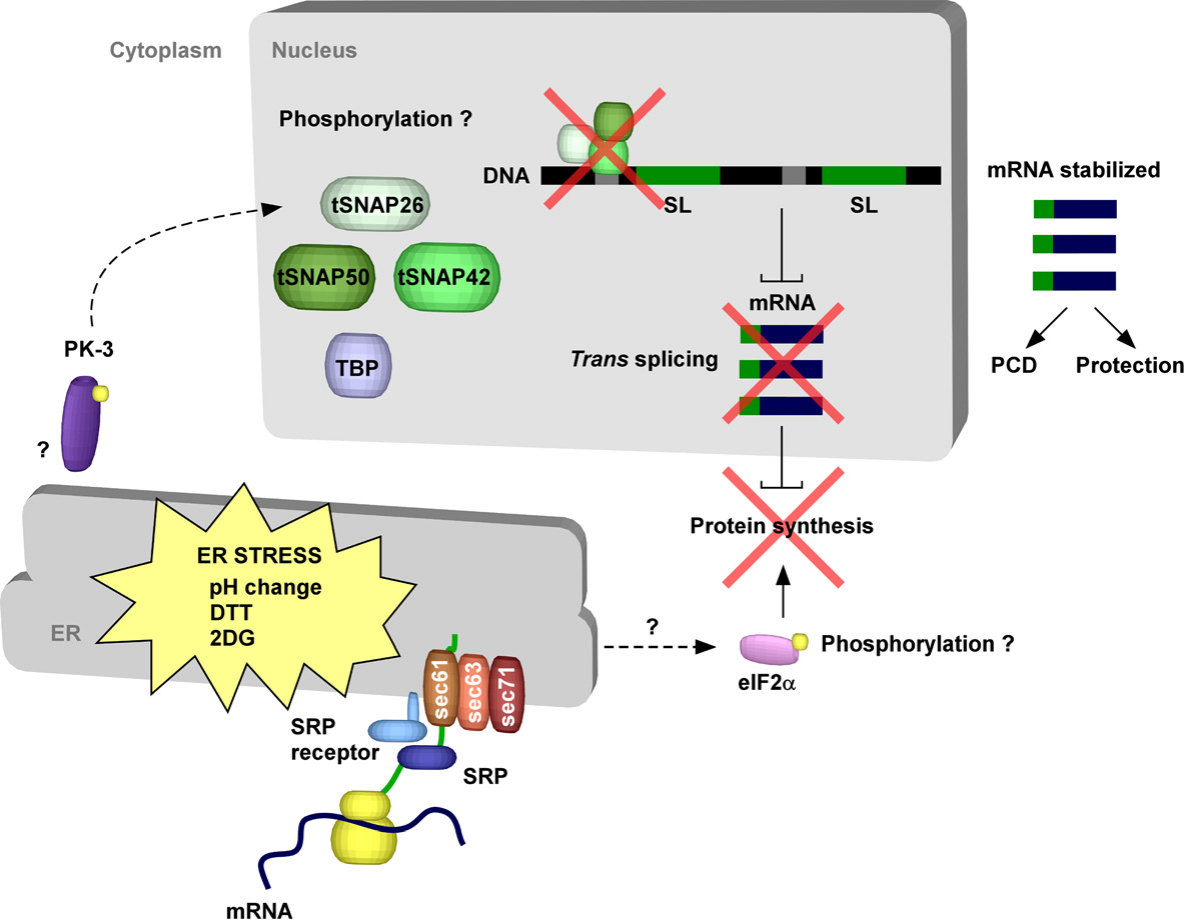
**The mechanism of SLS. In trypanosomes all mRNA are*trans*-spliced.** In this process, the exon or spliced leader is donated to the mRNA from a small RNA, the spliced leader RNA. The SL RNA is transcribed and assembled in a distinct nuclear site that was termed the SL factory [5], where the SL RNA is transcribed, modified and assembles with its binding protein. Under stress that perturbs the ER homeostasis such as blocking translocation of proteins across the ER membrane (via RNAi silencing of SRP receptor, SEC61, or SEC63) or by prolonged exposure to chemicals such as DTT and 2DG or under drastic pH changes, the SLS pathway is induced. The hallmarks of SLS are shut-off in SL RNA transcription due to the inability of tSNA42 to bind to the SL RNA promoter, leading to the accumulation of the tSNAP42 in the nucleus. The shut-off of SL RNA transcription leads to marked reduction in mRNA production and to induction of apoptosis. One key kinase in this pathway is PK-3, a serine-threonine kinase that transmits the signal from the ER to the nucleus. Despite the fact that *trans*-splicing is inhibited during SLS, the level of certain mRNAs is increased. These mRNAs may lead the synthesis of proteins that are essential for executing SLS.

Secretory proteins or membrane proteins need to traverse the ER to reach their final destination. Two pathways exist to execute this mission, the co-translational pathway mediated by the signal recognition particle (SRP) and post-translational route utilizing chaperones [55]. In the co-translational translocation pathway, the signal-peptide or the transmembrane domain is recognized by the SRP; the ribosome-nascent chain-SRP then binds to the membrane via the SRP receptor, and after SRP release, the translating ribosomes interacts with the translocon, and the protein is co-translationally translocated [55] (Figure 3).

The trypanosome factors belonging to these pathways and the ones relevant to this review are summarized in Table 1. RNAi silencing of the signal recognition protein SRP54 in *T. brucei* showed that SRP is essential for the survival of the parasites. Under SRP54 depletion, signal-peptide containing proteins traversed the ER membrane but were mislocalized and formed megavesicles that are reminiscent of autophagosomes [56-58]. The results indicated the post-translational pathway must operate in these parasites to enable protein translocation under SRP depletion. Indeed, RNAi silencing of a SEC71, a factor that was shown to mediate post-translational translocation across the ER in yeast, resulted in translocation defects but under these conditions proteins traversed the ER via the SRP pathway [8]. Only the translocation of glycosylphosphatidylinositol (GPI)-anchored proteins, the most abundant surface proteins of the parasite, was severely impaired in SEC71 depleted cells, suggesting that the GPI-anchored proteins are preferential substrates of the post-translational pathway [8]. On the other hand, polytopic membrane proteins were shown to absolutely require the SRP pathway [56].

**Table 1:** Factors involved in translocation of proteins across the ER membrane and their relationship to SLS

**Factor**	**SRP54**	**SRa**	**SEC61**	**SEC63**	**SEC71**
**Function**	One of the SRP proteins recognizes the signal peptide	SRP receptor localized on the ER membrane	The translocon localized in the ER membrane	Factor essential for co- and post translational translocation	Factor essential exclusively for post-translation translocation
**Translocation defects in RNAi-silenced cells**	Mislocalization of SP-proteins, no production of membrane proteins	Mislocalization of SP-proteins, no production of membrane proteins	Mislocalization of SP-proteins, no production of membrane proteins	Mislocalization of SP-proteins, no production of membrane proteins	Mislocalization of SP-proteins, reduction in GPI-anchored proteins, no effect on membrane proteins
**Induction of SLS**	**No**	**YES**	**YES**	**YES**	**NO**

The data is based on [2,8,9,56].

In the course of studying the cellular defects upon perturbing protein translocation across the ER, protein translocation defects were examined in cells silenced for the SRP receptor, SRα. Interestingly, although both SRα and SRP54-silenced cells share the same protein translocation defects [57,58], SRα but not SRP54 depletion caused the reduction of all mRNAs tested [2]. This reduction was a result of inhibition of *trans*-splicing, due to inhibition of SL RNA transcription since SL RNA is the donor of the SL exon present on all trypanosome mRNAs. Inhibition of SL RNA transcription was associated with the failure of tSANP42, a SL RNA-specific transcription factor to bind to the SL RNA promoter. The process was therefore termed SLS for spliced leader RNA silencing [2] (Figure 3).

SLS was initially discovered in SRα silenced cells but not in cells depleted for SRP proteins [2]. Later studies revealed that SLS is also induced in cells silenced for SEC63, a factor that is essential for both post- and co-translational translocation pathway, as well as in cells depleted for the ER translocon SEC61 (Table 1) [8,9].

### SLS is induced by chemicals that induce UPR in other eukaryotes

Since SLS was discovered under perturbations that interfere with translocation of proteins across the ER, thus inducing ER stress, we sought to examine if SLS is the trypanosome analogue of the conventional UPR response present in other eukaryotes. To examine if UPR exists in trypanosomes, but may be activated by a novel mechanism, which is not related to the UPR response in other eukaryotes, cells were exposed to the classical UPR inducer, the reducing agent dithiothreitol (DTT), and RNA was subjected to microarray analysis. Inspection of the up-regulated genes demonstrated the up-regulation of a distinct family of genes. These genes include genes involved in the core processes of UPR such as protein folding, degradation, translocation across the ER, protein sorting, redox balance, and lipid metabolism. Interestingly, other transcripts for genes involved in signal-transduction and RNA binding proteins were also increased. To examine if these alterations are reminiscent of changes that take place under UPR response of other organisms the microarray data was compared to data available for *Caenorhabditis elegansDrosophila melanogaster*, and *Homo sapiens*. The results of such analysis revealed that, in trypanosomes, the genes most strongly affected by DTT treatment are genes involved in protein secretion. Of additional interest is the finding that 35% of the genes whose level was reduced encode for proteins destined to traverse the ER i.e. proteins harboring either a signal-peptide or *trans-*membrane domain. These results are reminiscent of those in *Drosophila*, where DTT treatment elicits IRE1-dependent degradation of mRNA coding for proteins that need to traverse the ER [22], thus providing an additional mechanism to reduce the ER-load [9,22,23].

As indicated, trypanosomes lack transcriptional regulation, and although excessive alternative splicing was recently shown to exist in *T. brucei,* it is currently unknown how these events are regulated [48]. However, the most prevalent regulatory mechanism in trypanosomes is mRNA stability and preferential translation, which is mediated by the rich repertoire of RNA binding proteins [46,47]. It was therefore most reasonable to investigate the strongest branch of regulatory mechanisms, mRNA stability, regarding its potential role in regulating the level of mRNA under ER stress. Indeed, mRNA stability of selected mRNAs whose level was increased under DTT treatment was examined, and it was found that mRNA for the chaperone DNAJ, protein disulfide isomerase (PDI), thioredoxin, and syntaxin were increased; in contrast no change in stability of mRNAs whose level was unchanged during DTT treatment was detected, suggesting that mRNA stabilization is the mechanism that mediates the up-regulation of specific mRNAs during ER stress [9]. Indeed, PTB proteins that regulate both *trans*-splicing and mRNA stability were shown to regulate the stability of mRNAs coding for genes involved in protein trafficking [59]. Signaling pathway and additional RNA binding proteins are most probably involved in regulating the stability of mRNAs during ER stress. Recently, isobaric tag for relative and absolute quantitation (iTRAQ) analysis performed on SLS induced cells (by SEC63 silencing) revealed an increase in the level of the RNA binding proteins such as PTB2 and a protein containing a zinc finger domain (our unpublished data). These proteins may control the stability of mRNAs under ER stress. Under SLS, no mRNA is produced *de novo,* but up-regulation of specific mRNAs was observed by microarray analysis of SEC63 silenced cells (our unpublished results). The up-regulated mRNA may result from prolonged half-life during SLS, possibly as a result of elimination of RNA binding proteins that control mRNA stability. Thus, despite the major shutdown in mRNA production, there is a subset of mRNAs that are stabilized under SLS. These mRNAs may code for proteins that are essential to execute SLS. The signaling pathway induced under SLS may induce modifications on these RNA binding proteins thus changing their effect on mRNA stability under stress; i.e. such modifications may for instance make the protein bind the mRNA and stabilize it under stress or avoid its binding to destabilize the mRNA. Studies are in progress to examine the exact role of several RNA binding proteins on mRNA stability under normal ER stress, and SLS.

The ER-stress response in *T. brucei* was further studied by inspecting two parameters, which are the hallmark of UPR induction in eukaryotes, increased expression of the chaperone, BiP, and ER expansion. Our results demonstrate that BiP is increased in both stages of the parasites, procyclic and bloodstream forms, following treatment with 4 mM DTT and 20 mM of deoxy-glucose, which inhibits glycosylation and affects ATP production. The increase in BiP level could be the result of both stabilization of mRNA as well as preferential translation of the protein under stress. In addition, treatment of the cell with DTT leads to ER expansion and accumulation of aggregates within the expanded ER. These data support the notion that trypanosomes react to ER stress similarly to other organisms [9].

However, prolonged ER stress induced by DTT induces the SLS pathway, as demonstrated by the reduction of the SL RNA and by the accumulation of tSNAP42 in the nucleus. While cells can recover from treatment with DTT for up to 60 min, long treatments are irreversible and the cells die. Since BiP induction occurs before SLS induction, these results suggest that trypanosomes first activate the ER stress-response, and only when the stress is persistent is SLS induced [9]. A recent study also investigated the changes of *T. brucei* transcriptome during development, as well as in response to tunicamycin or DTT treatments in the bloodstream form trypanosomes. The study reached the conclusion that only limited changes take place in response to ER stress. The same study also failed to detect changes in BiP, which led the researchers to conclude that UPR might not exist in trypanosomes. It was argued, as mentioned above, that these parasites live under homeostatic conditions, especially in the mammalian host and therefore have no need for a response to environmental stress [50]. The discrepancy in the phenotype observed under DTT treatment may reflect differences in the DTT concentration used in these two studies.

One of the most intriguing questions is how in the absence of IRE1 and XBP1, the signal is transmitted from the trypanosome ER to the nucleus to induce SLS. Surprisingly, trypanosomes possess three homologues that resemble eIF2 kinases, termed TbIF2K1-K3 [60]. As opposed to yeast, which lack PERK homologues, one of these kinases, TbIF2K2, carries a transmembrane domain similar to PERK. This protein phosphorylates the trypanosome eIF2α on Thr^169^, which is homologous to Ser^51^ of other eukaryotes. However, this kinase is localized to the flagellar pocket of the parasite [60]. No change in the shutdown of protein synthesis was obtained as a result of DTT treatment in cells silenced for this factor by RNAi (our unpublished data). However, we have recently identified a kinase (PK-3) whose silencing abolished the SLS response. Cell silenced for PK-3 and SEC63 show, as expected, perturbations in protein translocation, but these cells fail to shut-off SL RNA transcription and to accumulate tSNAP42 (manuscript in preparation). Experiments are in progress to understand how this kinase associates with the ER membrane, which proteins interact with this kinase and how the signal is transmitted from the ER to the nucleus. Another open mechanistic question is what are the changes and the modification to the SL RNA transcription complex during SLS. Purification of the SL RNA transcription complex from SEC63-silenced cells revealed changes in the level of certain factors, but no modification was detected on tSNAP42 that would explain its failure to bind to DNA. However, another factor involved in SL RNA transcription was shown to be specifically phosphorylated under SLS (manuscript in preparation). This data and the involvement of PK-3 in SLS signaling suggest that SLS signaling is mediated by a phosphorylation cascade (Figure 3).

### SLS is a PCD pathway

Apoptosis was shown to take place under prolonged UPR. We therefore sought to examine if SLS induction leads to apoptosis. Apoptosis describes a process consisting of the controlled removal of cells from an organism or a population. Apoptosis is associated with distinct cellular changes including rounding-up of cells, chromatin condensation, DNA fragmentation, and membrane flipping exposing phosphatidyl-serine [61,62]. Apoptosis in metazoa is triggered by signals coming either from the outside (extrinsic) or from inside (intrinsic) that lead to limited proteolysis by caspases, which eventually causes cell disruption without inducing inflammation. In contrast, necrotic cells swell, then burst releasing compounds that cause inflammation. However, it was recently realized that the distinction between necrosis and apoptosis might be somewhat artificial. Moreover, apoptotic cells show signs of necrosis at the end of the death process [63]. Caspases activation was always considered as prerequisite of apoptosis. However, forms of caspase-independent apoptosis were subsequently described [64].

The situation in protozoa regarding apoptosis is confusing since apoptosis requires the activity of caspases, and these are absent in trypanosomes [65]. In addition, there was a lengthy debate if true apoptosis can occur in unicellular protozoa. However, as discussed previously [65] and more recently [66], protozoan parasites can be considered as a community that has an “interest” in controlling cell density. A carefully regulated infection can increase the chances for a sustained infection and efficient transmission to the next host. Support for this notion came from the observation that ‘stumpy form’ trypanosomes, which are the non-dividing form of the parasite present in the bloodstream, secrete prostaglandin D_2_ (PGD_2_), leading to PCD of the stumpy form [67]. The slender form is the dividing bloodstream parasite that maintains persistent parasitemia. The size of the population inside the mammalian host is controlled under infection by the effect of a factor secreted from the slender form that stimulates transformation from slender to stumpy. Thus, the slender form secretes the stumpy induction factor (SIF), which induces differentiation to the stumpy form. The stumpy form responds to PGD_2_ with PCD. The number of stumpy parasites thus decreases as a result of PCD, but will be replaced and is kept constant because of oscillating parasitemia [65]. Thus, the altruistic death of the stumpy form ensures a persistent infection. Other cases where PCD was described in the trypanosomatid family were shown to occur under different stresses such as heat shock, reactive oxygen species (ROS), anti-parasitic drugs, starvation, and following binding of antibodies and complement (recently reviewed) in [68].

SLS most closely resembles the “physiological” apoptosis-like state induced by endogenous prostaglandins, which is used to control the size of the population to maintain sustained infection [67,69]. SLS accelerates cell death, rapidly eliminating unfit organisms from the population. The apoptosis-like cell death of SLS-induced cells is a controlled pathway of destruction that occurs without liberation of harmful enzymes, like lysosomal hydrolases or even cell components that are released from dying cells and can induce inflammation in the host. The altruistic death of the sub-population of these cells is a beneficial strategy of the parasite to quickly eliminate the unfit cells, without damaging the entire population, thereby increasing the chances of survival within the host.

It was suggested that protozoan parasites, including African trypanosomes, perform a caspase-independent form of apoptosis. A recent review summarized the data supporting the appearance of apoptotic markers in parasites [70], and an additional review summarized the parasite functions that are necessary to execute apoptosis [68].

The major argument against the presence of apoptosis in trypanosomes is the absence of caspases [65]. However, the classical caspases might be replaced in trypanosomes by other proteases. The iTRAQ analysis of SLS-induced cells revealed an increase in the level of calpain-like cysteine peptidase. Co-silencing of this protease in SEC63 silenced cells abolished SLS and the apoptosis-associated with SLS but induced a quick necrotic death, suggesting the role of the family of these proteases in the apoptosis induced by SLS (manuscript in preparation).

SLS induction is accompanied by several physiological effects that are hallmarks of apoptosis such as: increase in cytoplasmic Ca^2+^, exposure of phosphatidyl serines, mitochondria depolarization, and production of reactive oxygen species (ROS). In addition, SLS-induced cells show classical DNA laddering, and DNA fragmentation that can be observed by the TUNEL assay or simply by examining the sub-G1 population [9].

One can envision a mechanism by which ER stress induces imbalance of Ca^2+^ homeostasis. Trypanosomes, like other eukaryotes, maintain a low intracellular level of free Ca^2+^. Several cellular compartments have the ability to transport Ca^2+^ in an energy dependent manner, including the plasma membrane, ER, mitochondrion and the acidocalcisome [71]. In trypanosomes, the mitochondrion maintains a low resting level of [Ca^2+^, but transiently accumulates large quantities of Ca^2+^ from the cytoplasm following Ca^2+^ influx across the plasma membrane or after release from the acidocalcisome [71]. Indeed, death in *T. brucei* was shown to be associated with changes in the ability of mitochondrion to modulate [Ca^2+^ levels. Such imbalance was also described in mammalian cells under UPR, leading to apoptosis [72]. The increase in cytoplasmic Ca^2+^ is most probably due to leakage from the malfunctioning ER, resulting from loss of the ER’s capacity to store Ca^2+^. Several causes might be responsible for the increase in cytoplasmic [Ca^2+^ including reduced levels of calreticulin, thus reducing the capacity to bind Ca^2+^ within the ER. In addition, reduced levels of ER-resident SERCA calcium pumps and acidocalcisome Ca^2+^ transporters may also lead to an increase in cytoplasmic Ca^2+^. Since SLS induced cells are defective in biogenesis of both signal-peptide containing proteins and polytypic membrane proteins, and these three proteins belong to this family, it explains how the ER translocation defects cause the perturbations in Ca^2+^ homeostasis.

In eukaryotes, Ca^2+^ from the ER or cytoplasm moves to the mitochondrial outer membrane through voltage dependent ion channels (VDAC) [73]. This leads to induced opening of the mitochondrial permeability transition pore (PTP) resulting in matrix swelling. Such changes cause the rupture of the outer membrane of the mitochondria, and release of apoptotic factors [74]. The rise in the mitochondrial Ca^2+^ stimulates the generation of ROS, and the opening of PTP causes dissipation of the mitochondrial outer membrane potential (ΨΨm), as was observed in SLS-induced cells. Thus, ER translocation leading to changes in Ca^2+^ homoeostasis may be sufficient to induce the death in trypanosomes.

If so, why is SLS induced, and why is this pathway not induced under SRP depletion or depletion of the post-translation translocation pathway? It was proposed that SLS might speed up the death process. SLS is induced when the response to ER stress fails to restore homeostasis, and it resembles apoptosis that takes place in mammalian cells under persistent ER stress [9].

Induction of SLS might be analogous to apoptosis induced by persistent UPR response. As mentioned above, in metazoa, a very complex and delicate system exists to control the decision between the protective and the destructive branches of the UPR response. This decision is for instance controlled by eIF2α phosphorylation; PERK activates the phosphorylation causing a shut-off of protein synthesis, but this phosphorylation is gradually inactivated by phosphatases such as GADD34 that liberate active eIF2α [43]. However, so far, our studies failed to detect mechanism analogous to eIF2α phosphorylation. Recently, evidence accumulated in the trypanosome field for the regulation by the phosphorylation of eIF2α. It was demonstrated that eIF2α phosphorylation is important for the intracellular differentiation of *Leishmania.* A *Leishmania* mutant that has impaired eIF2α phosphorylation during ER stress showed delayed differentiation into amastigotes grown axenically [75]. Recently, and using an antibody that recognizes the Thr^169^ that undergoes phosphorylation in trypanosomatids, as well as by examining the phenotype of mutants where Thr^169^ was substituted by alanine, it was demonstrated that the eIF2α pathway participates in the adaptive response of *T. cruzi* to nutritional stress, contributing to parasite differentiation to disease-causing metacyclic trypomastigotes [76]. iTRAQ analysis failed to detect the phosphorylation of trypanosome eIF2α in SLS-induced cells.

Moreover, tagging eIF2α and examining its modification during SLS failed to detect any changes in protein migration (our unpublished data). Interestingly, heat-shock in *T. brucei* causes polysome collapse and translational shut-off independently of eIF2α phosphorylation, which takes place during the heat-shock response in other eukaryotes [77]. Studies are in progress to use the anti-Thr^169^ antibodies to examine whether or not eIF2α undergoes phosphorylation under SLS.

### SLS and autophagy

As mentioned above, targets of the UPR include chaperones and biosynthetic enzymes for the synthesis phospholipids to expand the ER and thereby dilute the hazardous misfolded proteins. In addition, the accumulation of misfolded proteins also leads to activation of ER-associated protein degradation (ERAD), which mediates retrograde translocation of misfolded proteins into the cytosol for degradation by the proteasome [25]. Misfolded proteins from the ER, or proteins that fail to traverse the ER may utilize an alternative pathway for protein degradation, known as autophagy. Many of the autophagic factors were shown to be UPR target genes that are crucial for survival under ER stress [31]. Indeed, under ER stress, ER membranes were shown to undergo autophagy by a process known as ER-phagy [78]. The main purpose of this process is to sequester the damaged ER. Autophagy is also observed in trypanosomes during differentiation from the bloodstream form to procyclics [79]. The autophagy observed under differentiation is controlled and is terminated following morphological remodeling.

In both mammals and yeast, autophagosomes are formed by two different pathways; one involves ATG8, and the other ATG12 and ATG5. In *T. brucei,* three ATG homologues were found, ATG8.1, ATG8.2 and ATG8.3. ATG8.2 contains a C-terminal extension and is the most closely related to the protein present in higher eukaryotes [80].

Most of the functional information on this pathway comes from studies in *Leishmania* showing the existence of ATG5, ATG10 and ATG12 homologues that complement yeast deletion strains [81]. It is currently unknown if autophagy is activated and is used for identical biological functions in *Leishmania* and *T. brucei*[80]. Although trypanosomes possess an ER stress response, as argued above, this process might not be robust enough to deal with the catastrophe imposed on the cells by blocking of entry of proteins to the ER. Autophagy is most probably induced in these cells to remove the dilated ER including the misfolded proteins. Indeed, the induction of SLS triggers the formation of autophagosomes that were visualized both by using ATG8.2-YFP tagged parasites but also by transmission electron microscopy [9]. Autophagy might not be specific to SLS-induced cells but may also be utilized in cells depleted for SRP, since mega-vesicles carrying mislocalized signal-peptide containing proteins were shown to accumulate under SRP54 depletion [57]. The autophagy induced under these conditions might be solely to protect the cell from the deleterious effects of the accumulation of proteins on the ER membrane. Thus, this type of autophagy might be different from the process that is induced as part of differentiation, or under amino acid starvation. If induction of UPR and autophagy cannot alleviate the cell from the major catastrophe, then SLS is induced. TOR kinase was shown to regulate the balance between protein synthesis and degradation via autophagy. Like many eukaryotes, *T. brucei* possess two TOR kinases, TOR1 and TOR2. TOR1 knockdown triggers the appearance of autophagic vesicles. Its depletion causes morphological changes such as abnormal appearance of the ER, and formation of membrane whorls similar to those that appear in eukaryotes upon TOR1 inhibition [82,83]. Interestingly, co-silencing of TOR1 with SEC63 did not have any effect on SLS, suggesting that this TOR is not involved in SLS signaling (our unpublished results).

## Conclusions

Trypanosomes possess a UPR-like response despite the lack of the transcriptional-based machinery that conducts this process in other eukaryotes. The trypanosome UPR-response is regulated in a manner similar to the heat-shock response in these organisms by stabilizing the mRNAs which are essential to cope with the ER stress [46]. ER stress also induces autophagy, as was demonstrated by the formation of autophagosomes containing ATG8.2 [9]. However, when the ER stress is prolonged by continued exposure to reducing agents, exposure to extreme pH, or blocking entry to the ER by depletion of ER translocation factors (SRP receptor, SEC63 or SEC61), SLS is induced. So far, SLS was demonstrated in *T. brucei,* and the next challenge is to determine if this mechanism is shared among all trypanosomatid species. The two missing links in understanding the mechanism of SLS are: (1) identification of the signaling pathway that senses the ER stress and transmits the signal to the nucleus, and (2) understanding the molecular events underlying the shut-off of SL RNA transcription. Recently, we identified an essential kinase (PK-3) whose presence is required for executing SLS. This handle on SLS signaling should lead to discovery of the entire signal transduction pathway. The mechanism underlying SL RNA transcription shut-off remains under investigation, but so far, our data suggest that the mechanism does not involve post-translational modification on tSNAP42. As soon as the signaling pathway from the ER to the nucleus is fully understood, it will be possible to examine if the shut-off of SL RNA transcription can be reversed. Inducing SLS during infection could offer a powerful means to control trypanosomal diseases such as sleeping sickness. Thus, SLS can offer a novel drug target. Small molecules that can activate SLS, thereby leading to suicide of the parasite could be safe and effective drugs to fight the devastating diseases caused by these parasites.

## Abbreviations

ER, endoplasmic reticulum; UPR, unfolded protein response; SL RNA, spliced leader RNA; SLS, spliced leader RNA silencing; PCD, programmed cell death; PK, protein kinases; SRP, signal recognition particle; SP, signal peptide; PERK, PKR-like ER kinase; TOR, target of rapamycin; iTRAQ, Isobaric tag for relative and absolute quantitation; IRE1, Inositol requirement; ATF, activating transcription factor; CHOP, C/EBP-homologous protein; eIF2, eukaryotic initiation factor; ERAD, ER-associated protein degradation; 2DG, 2-deoxy glucose; tSNAP, trypanosome homologue to snRNA activating protein complex; SIF, stumpy induction factor; PTB, polypyrimidine tract binding; TRAF, tumor necrosis factor receptor; JNK, JUN-amino-terminal kinase; PDI, protein disulfide isomerase.

## Competing interests

The author declares that she has no competing interests.

## Author contribution

This manuscript was written by SM, who engaged in discussions and consultations with Prof. Michael Duszenko, University of Tubingen Germany, while writing this review. SM read and approved the final manuscript.

## Author details

The Mina and Everard Goodman Faculty of Life Sciences, and Advanced Materials and Nanotechnology Institute, Bar-Ilan University, Ramat-Gan 52900 Israel
